# Rare Extra‐Axial Chordoma of the Anteromedial Thigh: A Case Report

**DOI:** 10.1002/ccr3.70341

**Published:** 2025-04-22

**Authors:** Meagan E. Tibbo, Nishad Mysore, Jean Jose, Andrew E. Rosenberg, H. Thomas Temple

**Affiliations:** ^1^ Department of Orthopedic Surgery Mayo Clinic Phoenix Arizona USA; ^2^ Division of Musculoskeletal Radiology, Department of Radiology University of Miami Miami Florida USA; ^3^ Department of Pathology and Laboratory Medicine University of Miami Miami Florida USA

**Keywords:** anteromedial thigh chordoma, brachyury, chordoma, extra‐axial chordoma, soft tissue chordoma

## Abstract

Chordomas are rare notochordal malignancies typically occurring in the axial skeleton. Less than 100 extra‐axial chordomas have been reported. We describe a unique chordoma of the anteromedial thigh. The clinical presentation of extra‐axial chordomas is highly variable; thus, recognizing this entity in the differential diagnoses of extra‐axial bone/soft tissue tumors is important.

## 1 Introduction

Conventional chordomas are rare malignant tumors that arise from remnant notochordal tissue and typically arise in the axial skeleton. Half of all such tumors are located in the sacrococcygeal area, whereas the rest predominantly affect the sphenooccipital region or mobile vertebral segments [1]. Rarer still are extra‐axial chordomas. On review of the literature, there were less than 100 total documented cases [2, 3]. Histologically and immunohistochemically, extra‐axial chordomas share many features with conventional axial chordomas. Regardless of primary location, conventional chordomas consist of epithelial cells with vacuolated and eosinophilic cytoplasm in a myxoid background. Expression of keratins is also characteristic [4]. Brachyury, an essential transcription factor in the formation of the notochord, is currently the most accurate assay for diagnosing both extra‐ and axial chordomas [1]. Once the diagnosis of an extra‐axial chordoma has been made, a discussion of the next steps in patient care can prove challenging. Because extra‐axial chordoma is so rare, an accurate assessment of survival, metastatic risk, and recurrence is difficult. However, the mainstays of treatment remain combination radiotherapy and surgical resection with wide margins. Radiotherapy alone, although efficacious, is not sufficient to achieve cure [5]. To the best of our knowledge, there have been no reports of an intra‐articular, extra‐synovial soft tissue chordoma involving the appendicular skeleton. Therefore, we present a case of an extra‐axial chordoma along with our approach to diagnosis and treatment. Written patient consent for the submission of this report has been obtained.

## 2 Case History/Examination

The patient is an otherwise healthy, 56‐year‐old male who presented to our office with a large and painless right anterior thigh mass. In retrospect, he felt that the mass had been present for approximately 1 year, and he did not notice an appreciable change in its size. It was identified incidentally on radiographs obtained as a part of an evaluation for osteoarthritic bilateral knee pain. The patient had no history of trauma, constitutional symptoms, or thigh pain. He had no personal or family history of malignancy. Clinical examination revealed a large, painless, immobile, and firm mass in the anteromedial aspect of the mid‐distal thigh. Knee range of motion was unaffected, and there was no inguinal lymphadenopathy. The patient's neurovascular status was intact, and the remainder of his physical examination was unremarkable.

## 3 Conclusions and Results

### 3.1 Imaging Features

**FIGURE 1:**
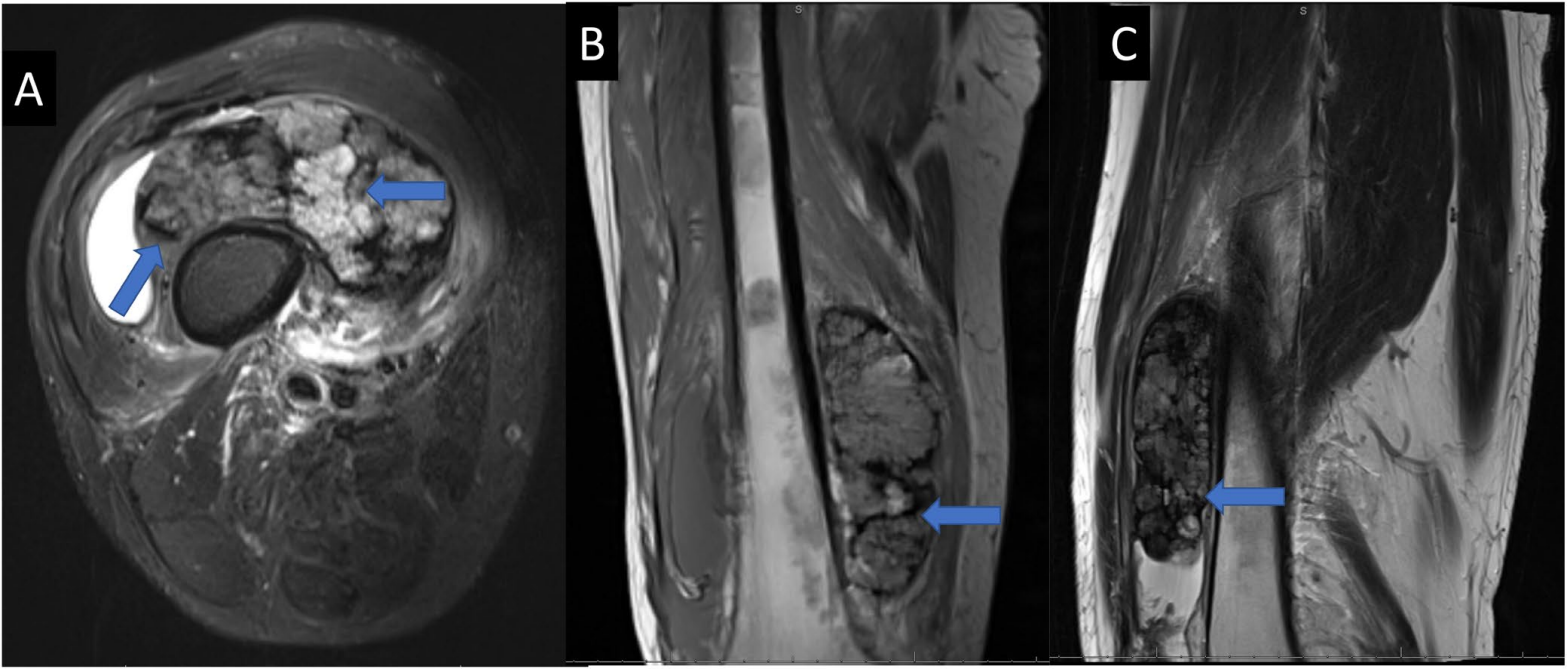
Axial T2 fat suppressed (A), coronal T1 (B) and sagittal proton density. (C) MRI demonstrates a solid extra‐skeletal extraarticular lobulated soft tissue mass, with heterogenous T1 and T2 signal intensity adjacent to the femoral diaphysis, with mild peritumoral edema. There is no evidence of cortical or medullary osseous involvement. The lesion contains internal linear and curvilinear areas of low signal intensity corresponding to hemosiderin deposition and fibrous septa (blue arrows).

Presenting radiographs demonstrated medial joint space narrowing and valgus knee alignment as well as a large non‐mineralized radiodense soft tissue mass in the medial thigh. Magnetic resonance imaging (Figure 1) demonstrated a massive joint effusion and a 14 cm, well‐circumscribed, heterogeneous mass deep to the vastus medialis and intermedius. The mass appeared to be intra‐articular and extra‐synovial. Signal characteristics included a T2 hypointense periphery with a lobular, mixed hypo‐ and hyperintense internal portion. There was no underlying osseous involvement. Because of the size and heterogeneity of the lesion, a core needle biopsy was obtained. The histopathologic features were interpreted as a conventional, extra‐axial chordoma.

### 3.2 Staging and Treatment

Staging CT of the chest, abdomen, and pelvis demonstrated several < 3 mm calcified lung nodules, prominent obturator, external iliac, and inguinal lymph nodes, as well as hypodensities in the liver thought initially to represent metastatic disease. MRI of the spinal axis did not demonstrate features reminiscent of chordoma.

Following discussion at our multi‐disciplinary tumor board, the decision was made to proceed with 30Gy of neo‐adjuvant hypofractionated (five fractions) stereotactic body radiation therapy (SBRT) followed by surgical resection. Neo‐adjuvant radiation therapy was utilized on the basis of our institutional preference for a lower overall radiation dose and decreased postoperative fibrosis.

The timeframe between radiation therapy and surgical resection was approximately 3 weeks, which is our standard protocol for the majority of soft tissue sarcoma cases. After ensuring appropriate healing of the skin, wide local excision was carried out. Surgical margins were obtained from the specimen, and a uniform residual (R) tumor classification of R0, indicating no residual tumor, minimal distance between the tumor and resection margin, and margins ≥ 1 mm were confirmed by pathology [6]. Wide surgical excision did not require sacrifice of the extensor mechanism, and as such, a free functional flap was not required. There was ample skin, fascia, and muscle tissue for primary wound closure.

### 3.3 Pathology

**FIGURE 2:**
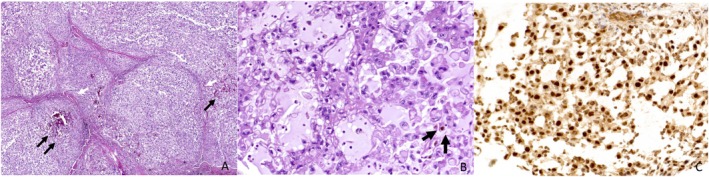
(A) Tumor is sub‐compartmentalized into nodules by fibrous septa (black arrows) and moderately cellular. (B) The polyhedral tumor cells are arranged in cords and aggregates. They have abundant eosinophilic cytoplasm with clear vacuoles. (C) The tumor cells show diffuse strong nuclear staining for brachyury.

**FIGURE 3:**
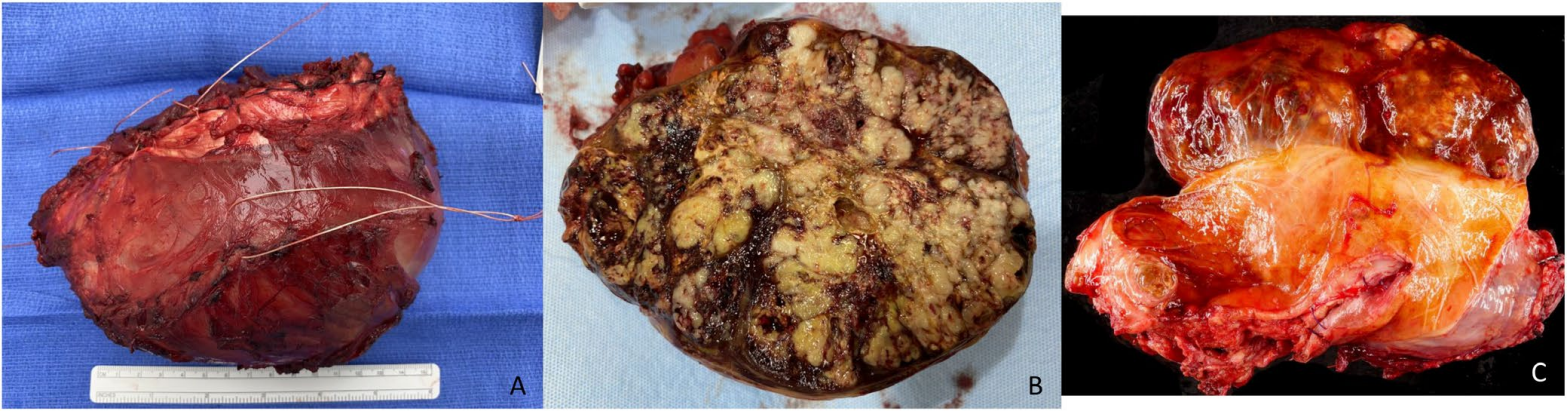
(A) Resection specimen. (B) Gross pathology after cross‐sectioning. (C) Extra‐axial chordoma bulging the overlying hemosiderin‐stained synovium. The mass is multinodular and red‐brown and tan. On the deep portion of the mass, the tumor is covered by muscle.

**FIGURE 4:**
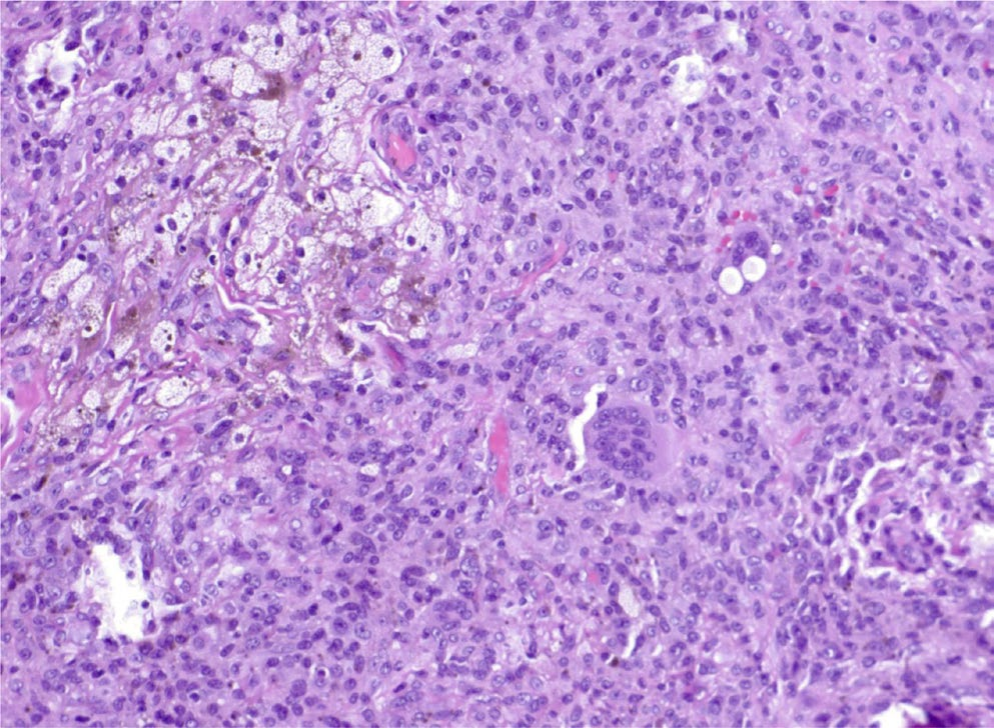
Tenosynovial giant cell tumor is hypercellular and composed of sheets of polyhedral cells admixed with foamy macrophages, hemosiderin deposits, and osteoclast‐type giant cells.

At the time of needle biopsy, aside from the usual histopathologic features of chordoma, tumor cells were positive for brachyury, keratin AE1/3, and focally positive for synaptophysin (Figure 2). Nuclear expression of INI1 was also deficient. The resection specimen and gross pathology image are found in Figure 3. Pathologic assessment of the specimen demonstrated a 12.3 cm mass with 10% necrosis and all margins negative for tumor. Interestingly, abundant, proliferative, and hemosiderin‐stained synovium, resected en bloc with the tumor, contained a focal 1.2 cm tenosynovial giant cell tumor (Figure 4).

### 3.4 Postoperative Course

**FIGURE 5:**
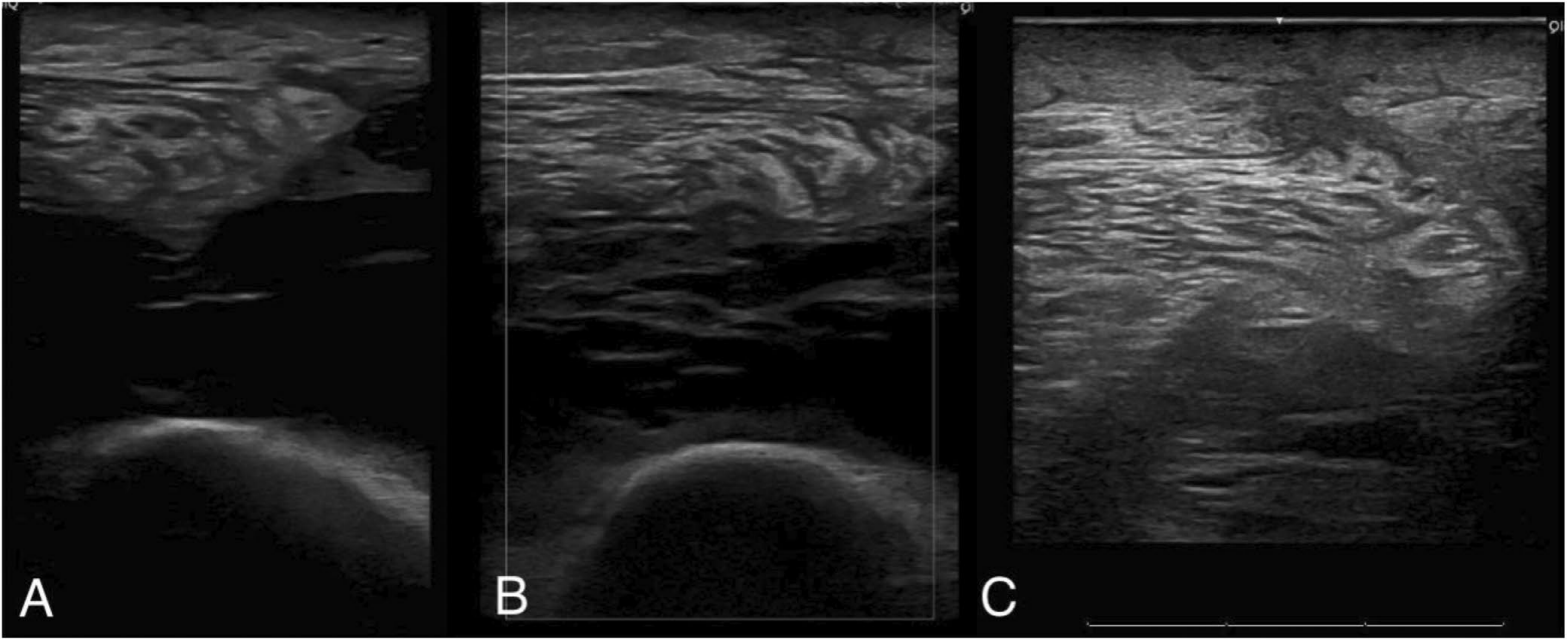
(A) Ultrasound done at postoperative follow‐up on August 10, 2023. (B) Ultrasound done at postoperative follow‐up on August 31, 2023. (C) Ultrasound done at postoperative follow‐up on November 13, 2023. Postoperative seroma is clearly visualized. Over the course of time, seroma resolution is visible, as is tissue regeneration.

The patient had an unremarkable postoperative course and was discharged home on the second postoperative day. The patient was followed using our standard surveillance protocol with an MRI of the local site and a CT scan of the chest every 3 months for the first 2 years after surgical resection. At 1 year postoperatively, the patient had no clinical or radiographic disease recurrence. He had no functional limitations and was not using assistive devices. He had a minimal knee effusion. Immediate postoperative ultrasound performed at bedside in the office showed residual seroma in the deep anterior thigh. Serial ultrasounds at routine follow‐up intervals indicated that the seroma significantly decreased from immediate postoperative imaging over the course of the next several months. Seroma aspiration is not our institution's routine standard of care if the patient remains asymptomatic. In this case, the patient had no symptomatic sequela from the seroma, and it was self‐resolving. (Figure 5).

## 4 Discussion

Due to the rarity of extra‐axial chordomas, the initial diagnosis was surprising, the prudent course of treatment was uncertain, and the prognosis is ostensibly unknown. Brachyury is routinely assayed as it is a transcription factor of notochordal tissue and is currently the most sensitive and specific finding ascribed to notochord tissue. Once suspicion of extra‐axial chordoma was corroborated with sufficient clinical, radiographic, and pathologic evidence, the planned treatment in this case combined a wide surgical resection and external beam radiotherapy. Surgical resection with wide margins remains the mainstay of treatment for chordoma. Multiple series have demonstrated that margin status correlates with survival in patients with chordoma [7] Philosophically speaking, the approach to chordoma treatment is to view these tumors as locally malignant lesions with metastatic potential. Thus, our approach mirrored the commonly utilized current treatment paradigm utilizing neo‐adjuvant radiation and surgery in an effort to decrease the risk of local recurrence post‐resection [7]. Radiotherapy alone was felt to be insufficient to achieve local disease control as it is deemed inadequate in patients with conventional axial chordoma [5]. Additionally, extra‐axial chordomas tend to be amenable to wide surgical resection, allowing for sufficient margins to be achieved, thereby making adjuvant radiotherapy a less attractive option [8].

In our case, the clinical presentation was non‐specific, with the physical examination demonstrating a firm, painless, and immobile mass in the anteromedial thigh that was either very slowly growing or stable in size for at least a year. This is not unlike the case reported by Blitzer et al. wherein an extra‐axial chordoma between the third and fourth metacarpals slowly increased in size for 7 years prior to becoming painful with activity [9]. Van Akkooi et al. described a patient with a painful dorsal hand mass [5]. Our patient's presentation differs from that of previously described extra‐axial chordomas of the lower extremities. Among three cases available in the current literature, all presented with tumors originating from bone or with osseous involvement and pain [8, 10]. According to the Blitzer et al. study, all documented brachyury‐positive extra‐axial chordomas that were localized to soft tissue occurred only in the hand [9]. Our case likely represents the first reported extra‐osseous extra‐axial soft tissue chordoma of the lower extremity.

Given the dearth of documented cases of extra‐axial chordoma, it is difficult to generate detailed image‐based diagnostic criteria, and definitive diagnosis requires careful histopathologic and immunohistochemical analysis. However, some similar/overlapping radiographic features can be seen (especially on MRI). Our patient's initial radiographs demonstrated a radiodense soft tissue mass with no internal mineralization or obvious osseous involvement. In general, radiographs are non‐specific and are useful for delineating mineralization or secondary bone involvement (erosion or medullary invasion); they are especially useful in cases arising in bone [8, 9, 10, 11, 12, 13]. However, MRI results demonstrate a lobulated mass with mixed signal intensity, especially on T2 weighted pulse sequences in a manner similar to that described in our case [8, 9, 10, 11, 12, 13].

Although a large midline sacral mass that is hypointense on T1 pulse weighted sequences and relatively uniformly hyperintense on corresponding T2 images is presumptive of a chordoma, corroborating histopathologic and immunohistochemical analysis is necessary to confirm the diagnosis. Extra‐skeletal chordomas typically appear on MRI as lobulated masses with well‐defined borders. They show heterogeneous signal intensity, with areas of both high and low signal on T1‐ and T2‐weighted images, depending on the presence and number of internal calcifications, hemorrhagic components, and necrotic areas within the tumor. Post‐contrast images demonstrate variable areas of enhancement, with some parts showing avid enhancement and other regions showing minimal or no enhancement. This tumor often exhibits internal fibrous septa with surrounding extracellular myxoid matrix, which may resemble myxoid chondrosarcoma. The brachyury assay, performed in all cases we identified in the literature, is highly specific for chordoma [1, 2, 4, 5, 8, 9, 10, 11, 12, 13]. Histologically, vacuolated epithelioid cells with eosinophilic cytoplasm or physaliphorous cells in a myxoid matrix background are also characteristic. The case presented herein provides additional data to suggest that a lobulated heterogeneous mass may be a characteristic, although non‐specific, of extra‐axial chordoma [1]. Tumor cells from our patient demonstrated brachyury, keratin AE1/3, and focally synaptophysin positive staining along with a loss of nuclear expression of INI1. Although the loss of INI1 is more commonly observed in poorly differentiated chordomas, a minority of conventional chordomas have been shown to lose INI1 expression as well. There seems to be a genetic correlation with the loss of INI1 expression in conventional chordomas as this can be caused by deletion of chromosome 22, heterologous deletion of INI1, mutation of INI1, or homozygous deletion—the latter of which is present in dedifferentiated chordomas [4, 14]. In a clinicopathological analysis of six cases by Righi et al. the same immunohistochemical markers were expressed (or attenuated in the case of INI1) [2]. Additionally, there is significant overlap of these particular molecular markers with most cases reviewed [1, 2, 4, 5, 8, 9, 10, 11, 12, 13]. One study sought to characterize clinicopathologic characteristics of extra‐axial chordoma as compared with conventional chordoma in a cohort of 86 cases. The authors found that there were no differences in the expression of several immunohistochemical markers, including brachyury, pancytokeratin, cytokeratin 19, and S‐100 [3]. They additionally attempted to quantify the prognostic value of the aforementioned markers and found that, apart from diagnostic utility in extra‐axial chordoma, there was a lack of predictive value. However, they did note a significant increase in CAM5.2 or vimentin expression among extra‐axial chordomas compared with their axial counterparts [3]. These data may prove useful when interpreting the phenotypic differences between extra‐axial chordomas and axial chordomas in the future.

Extra‐axial chordoma is a rare tumor and not suspected in patients with extra‐axial soft tissue masses. Moreover, the clinical presentation among patients with extra‐axial chordoma is highly variable, as are the radiographic findings. Therefore, it is important to consider and recognize the clinical, radiographic, and histopathologic features of this entity in the wide spectrum of extra‐axial bone and soft tissue lesions.

## Author Contributions

**Meagan E. Tibbo:** conceptualization, data curation, formal analysis, funding acquisition, investigation, methodology, project administration, resources, software, supervision, validation, visualization, writing – original draft, writing – review and editing. **Nishad Mysore:** data curation, writing – original draft, writing – review and editing. **Jean Jose:** conceptualization, data curation, formal analysis, funding acquisition, investigation, methodology, project administration, resources, software, supervision, validation, visualization, writing – original draft, writing – review and editing. **Andrew E. Rosenberg:** conceptualization, data curation, formal analysis, funding acquisition, investigation, methodology, project administration, resources, software, supervision, validation, visualization, writing – original draft, writing – review and editing. **H. Thomas Temple:** conceptualization, data curation, formal analysis, funding acquisition, investigation, methodology, project administration, resources, software, supervision, validation, visualization, writing – original draft, writing – review and editing.

## Consent

Written informed consent was obtained from the patient to publish this report in accordance with the journal's patient consent policy. A signed copy for publication of the case alongside accompanying images is held with the corresponding author and institution.

## Conflicts of Interest

The authors declare no conflicts of interest.

## Level of Evidence

Level V.

## Data Availability

Data available on request from the authors The data that support the findings of this study are available from the corresponding author upon reasonable request.
